# Evaluation of learning environment among Nursing undergraduates in state universities, Sri Lanka

**DOI:** 10.1186/s12912-021-00714-z

**Published:** 2021-10-09

**Authors:** Patalee Jayaweera, Abisheka Thilakarathne, Madushanka Ratnayaka, Tharangi Shashikala, Rushani Arachchige, Lahiru Sandaruwan Galgamuwa, Nimantha Karunathilaka, Thamara Amarasekara

**Affiliations:** 1 Department of Nursing and Midwifery, Faculty of Allied Health Sciences, General Sir John Kotelawala Defence University, Colombo, Sri Lanka; 2 Department of Parasitology, Faculty of Medicine, Sabaragamuwa University of Sri Lanka, Belihuloya, Sri Lanka; 3 Department of Nursing & Midwifery, Faculty of Allied Health Sciences, University of Sri Jayewardenepura, Nugegoda, Sri Lanka

**Keywords:** Evaluation, Learning Environment, Nursing Undergraduates, The Dundee Ready Educational Environment Measure, Sri Lanka, and State Universities

## Abstract

**Background:**

The learning environment is a vital part of the undergraduate curriculum which enable to delivery of quality education in the stipulated time. Therefore, this study aimed to evaluate the learning environment among BSc. Nursing undergraduates in Sri Lankan state universities.

**Methods:**

A descriptive cross-sectional study was conducted among 161 final year BSc. Nursing undergraduates in six state universities. Socio-demographic characteristics were collected using a self-administered questionnaire. The Dundee Ready Educational Environment Measure (DREEM) questionnaire was used to evaluate the learning environment in Perception of learning (SPL), Perceptions of teaching (SPT), Academic self-perceptions (SASP), Perceptions of the atmosphere (SPA), and Social self-perceptions (SSP). Based on the SPL, SPT, SASP, SPA, and SSP domains, the overall score of learning environment was ranged from 0 to 200 and then the overall score was classified into four categories such as poor (0–50), many problems (51–100), more positive than negative (101–150) and excellent (151–200). One-way Analysis of Variance (ANOVA) and t-test were used to determine the difference in the subscales and the overall scale.

**Results:**

The mean age of the students was 24.9 ± 0.9 years. The overall score of the learning environment was 127.1 ± 14.3. Student’s Perception of learning showed the highest mean score of 31.1 ± 3.9 while the social self-perception showed the lowest score (mean 16.4 ± 3.1). A significant group effect was observed in SPL and SPT subdomains among state universities while no significant group effect was observed in other subdomains. Furthermore, participating in extracurricular activities, travelling time to the faculty, and gender were observed as associated factors for the learning environment among BSc. Nursing undergraduates in state universities.

**Conclusions:**

Although the overall learning environment of BSc. Nursing undergraduates in state universities in Sri Lanka was within more positive than negative category, none of the university reaches to the excellent category. Therefore, each university should have improved their subdomains of learning environment to reach excellent category through addressing the gaps of curricular and extracurricular activities in the future.

## Background

The learning environment straightly connects to the attainment, happiness, fulfilment, and favorable outcome of the students and also leads to the quality of the educational program [1, 2]. The successful learning environment connects with the best results of the learning institute and it develops values, views, and professional performances of students [2, 3]. Furthermore, the learning environment is an inescapable part of the syllabus, influencing the association between students, techniques, assessments, and academic consequences [4]. Students’ Perception of the academic learning environment is related to their learning viewpoint and the learning result and also it is an excellent beginning to be looked into in nursing education [5]. Furthermore, the learning environment plays an essential character in association with students’ way of behaving, academic development, feeling of comfort and security during their degree program [2].

At present, a four-year degree program has been conducting for Bachelor of Science (BSc) in Nursing undergraduates by the University Grants Commission (UGC) in Sri Lanka, in five universities, follows; University of Sri- Jayewardenepura, University of Peradeniya, Eastern University, University of Jaffna and University of Ruhuna [6]. Furthermore, General Sir John Kotelawala Defence University has also been offering the same UGC accredited BSc. Nursing degree program under the Ministry of Defense. Additionally, the Open University of Sri Lanka has been offering a special BSc. Nursing degree program for registered nurses who are already qualified with a diploma in nursing from the Ministry of Health, Sri Lanka as a post-registration program [6].

The concept of learning environment describes as the conditions, external stimuli and forces which may be physical, social, as well as intellectual forces which challenge on the individual and influence students’ learning outcomes [7]. Furthermore, clinical learning environment plays a significant role in nursing education and the main attribute characteristics were physical space, psychosocial and interaction factors, organizational culture and, teaching and learning components [8]. Fernandes et al., [9] stated that age, gender and monthly income were associated with learning environment. The Dundee Ready Educational Environment Measure (DREEM) was developed by Dundee University, UK is used to measure the learning environment across the globe [10]. DREEM is a worldwide validated tool for measuring the learning environment in medical institutions [11]. Although there are several tools available to measure the medical learning environment, the DREEM was mainly used to assess the learning environment in the medical, dental, and nursing undergraduate program especially in Asia and Europe [10, 11]. The DREEM consists of the five subdomains to evaluate the learning environment: Perception of learning (SPL), Perceptions of teaching/instructors (SPT), Academic self-perceptions (SASP), Perceptions of the atmosphere (SPA), and Social self-perceptions (SSP). A higher DREEM score indicates a higher level of the learning environment.

The learning environment straightly impacts the learning process of nursing students [1]. Furthermore, the majority of universities have observed positive aspects of the learning environment while few had negative when delivering the nursing curricula [12]. However, two universities in Sri Lanka; the University of Ruhuna and Eastern University had been evaluated their learning environment in BSc. Nursing degree programs in the year 2012 and 2016 respectively and the findings revealed that both universities were classified in more positive than negative category [13, 14]. However, there was a paucity of data available in other degree programs in state universities. At present, all the BSc. Nursing degree programs are mature enough with facilities and staff and also successfully conducted at least one curriculum revision in recent two years. Therefore, it is a timely needed necessity to evaluate the learning environment of BSc. Nursing undergraduates in Sri Lankan state universities and compare the outcomes for better nursing education in Sri Lanka. Hence, this study aimed to evaluate the learning environment among BSc. Nursing undergraduates in Sri Lankan state universities.

## Methods

### Study setting and population

A descriptive cross-sectional study was conducted with all final year (4th Year) B.Sc. nursing undergraduates in six state universities during the period of August to November 2019. These universities were mentioned anonymously based on privacy and confidentially in arbitrarily order (A-F). The final year (fourth year) nursing undergraduates are more familiar with the delivered nursing curriculum, facilities, and teachers which provide substantial information of each degree program.

The convenient sampling method was used to collect data among all the fourth-year nursing undergraduates in each state university. The principal investigators (PIs) instructed all final year nursing undergraduates regarding the inclusion criteria for participants, and each nominated coordinator of the respective university referred the eligible participants to the PIs. Undergraduates who were studies in the final (fourth) year and successfully participated or completed up to their third-year final examination. Undergraduates who were undergone medical leave or absence during the data collection period in each university or did not provide their consent to participate in the study were excluded. Therefore, a total of 161 B.Sc. Nursing final year students studying in Sri Lankan state universities [A (n = 29), B (n = 20), C (n = 23), D (n = 29), E (n = 27) and F (n = 43)] were participated for this study.

### Instrument

Study data were collected using a self-prepared demographic and DREEM questionnaire.

Self-prepared demographic form.

The demographic data were collected from the self-administered demographic questionnaire that includes age, gender, type of living place, mode of transportation to university, the average time to reach university, engaging with part-time employment, participating in the additional professional course, and extracurricular activities.

DREEM Questionnaire.

The pretested (n = 10) original version of DREEM was administered to evaluate the learning environment. The original version of the DREEM questionnaire was developed by Sue Roff and her colleagues at Dundee University, UK in 1997 [7, 9]. The DREEM includes 50 items to determine the learning environment under the following sub-domains: SPL (12 items), SPT (11 items), SASP (8 items), SPA (12 items), and SSP (7 items) respectively. Each item score 0–4 on a 5-point Likert scale (4- strongly agree, 3-agree, 2-unsure, 1-disagree, 0-strongly disagree) while 10 items are negative statements and should be scored in a reverse manner. The maximum score of the DREEM questionnaire is 200. Each item with a mean score ≥ 3.5 was considered as are true positive points while those ≤ 2 mean score was problem areas. The total value between 0 and 50 was considered as very poor followed by 51–100 = many problems, 101–150 = more positive than negative, 151–200 = excellent. The content validity was performed in the original version of the DREEM by using Delphi techniques and was concluded that the DREEM is the universal diagnostic tool to evaluate the learning environment [10]. Furthermore, DREEM has been translated into many different native languages namely Arabic, Chinese, Japanese, Persian, Portuguese, Spanish, Swedish, Turkish, and Urdu [15, 16]. In the South Asian region, Pakistan was the only country that validated the original DREEM questionnaire into their native language and the internal consistency was ranged from 0.7 to 0.9 in the overall score of DREEM as well as all the sub-domains [16]. Similarly, Haque et al., [15] also revealed that the internal consistency of overall DREEM was 0.7.

### Ethical consideration

Ethical clearance to the study was obtained from the Ethical Review Committee, Faculty of Medicine, General Sir John Kotelawala Defence University, Sri Lanka. Furthermore, the written permissions were obtained from the Deans of respective faculties and Heads of the Department of Nursing and Midwifery of each university before collecting data. Before the implementation of the study, written informed consent was obtained after providing necessary information verbally and information sheet that included, subject right to withdraw from the study at any stage, potential risks, and benefits, protect the vulnerability, privacy, and confidentiality of the students. All data obtained were securely stored and were accessible only to the PIs and supervisors.

### Data collection

Before the commencement of the study, each Nursing student was informed of the purpose of the study. In addition, the procedure and the definitions of medical terms were explained. A reasonable time was given to complete the questionnaire. Completed socio-demographic and DREEM questionnaires were collected from 161 BSc. Nursing students.

### Statistical analysis

All collected data were entered into Microsoft EXCEL 2007 and transformed into the statistical package for social sciences (SPSS) software version 20. Normal distribution of the data was confirmed by the Shapiro-Wilk test and parametric tests were used for analysis. One-way Analysis of Variance (ANOVA) was used to determine the difference in overall and its subscales with state universities. Pearson correlation was applied to determine the association between subscales. Student t-test was used to determine the association with subscales across the selected socio-demographic variables. The significant level was taken as p < 0.05.

## Results

A total of 161 students with a mean of 24.9 ± 0.9 years have participated in the study in six state universities. The overall score for the perception of the learning environment was 127.1 ± 14.3.

Among the five subscales, students’ perception of learning showed the highest mean score of 31.1 ± 3.9 while the social self-perception showed the lowest score (mean 16.4 ± 3.1). The overall score of DREEM and its subscales means and standard deviation are depicted in Table 1.

**Table. 1:** Scores of the overall perception of the learning environment and its subscales (*n* = 161)

Subdomains	Maximum	Mean	Range	Interpretation
	value	(SD)	(min.-max.)	
Perception of learning (SPL)	48	31.1 (3.9)	16–40	More positive perception
Perceptions of teaching (SPT)	44	26.5 (4.3)	15–40	Moving in the right direction
Academic self-perceptions (SASP)	32	22.8 (3.3)	13–32	Feeling more on the positive
Perceptions of the atmosphere (SPA)	48	30.1 (5.0)	9–45	More positive attitude
Social self-perceptions (SSP)	28	16.4 (3.1)	3–22	Not too bad
Total score	200	127.1 (14.3)	72–173	More positive than negative

SD: Standard deviations

The overall mean score of DREEM in the university A was 126.1 ± 10.4 followed by B was 123.3 ± 12.9, C was 128.3 ± 14.3, D was 132.3 ± 12.3, E was 126.5 ± 15.5 and F was 127.1 ± 11.4. The overall mean scores indicated that all universities were within a more positive than a negative learning environment. University D was significantly high in SPL and SPT compared to other universities. However, SASP, SPA, and SSP was not significantly associated with different universities (Table 2).

**Table. 2:** Comparison of learning environment subdomains with state universities

Sub-domains	Universities (mean ± SD)						
	A	B	C	D	E	F	p-value
Perception of learning (SPL)	29.5 (2.7)	31.3 (4.8)	32.1 (4.3)	33.6 (3.2)	30.0 (4.6)	31.2 (3.3)	0.007
Perceptions of teaching (SPT)	27.6 (2.9)	25.5 (6.0)	26.4 (3.2)	28.5 (3.5)	28.0 (4.6)	24.6 (4.0)	0.002
Academic self-perceptions (SASP)	22.2 (3.3)	22.1 (3.5)	23.0 (3.3)	23.0 (2.1)	22.4 (3.3)	23.5 (3.6)	0.518
Perceptions of the atmosphere (SPA)	30.9 (4.3)	28.6 (4.6)	30.2 (5.4)	30.8 (4.7)	29.3 (4.9)	30.6 (3.8)	0.530
Social self-perceptions (SSP)	15.9 (2.4)	15.8 (4.4)	16.6 (2.9)	16.2 (3.5)	16.7 (2.5)	16.9 (3.0)	0.661
Total score	126.1 (10.4)	123.3 (12.9)	128.3 (14.3)	132.3 (12.3)	126.5 (15.5)	127.1 (11.4)	0.527

Pearson correlation coefficients were calculated in Table 3. There was a significant positive correlation in subscales (p < 0.05) (Table 3). Furthermore, concerning problem areas of perception of learning environment among participants was depicted in Table 4. Mean scores of < 2.00 are reflective of problem areas in the educational environment. The following items had a mean score of < 2.00 from students. ‘The teaching over emphasize factual learning,’ ‘The teachers ridicule the students,’ ‘The teachers are authoritarian,’ ‘The teachers provide constructive criticism here,’ ‘The teachers get angry in class,’ ‘I find the experience disappointing,’ ‘I am too tired to enjoy this course’ and ‘I seldom feel lonely’(Table 4).

**Table. 3:** Pearson correlation with subscales

Subscale		SPL	SPT	SASP	SPA	SSP
SPL	Correlation coefficient	-	0.315	0.476	0.465	0.325
	p-value	-	< 0.001	< 0.001	< 0.001	< 0.001
SPT	Correlation coefficient	0.315	-	0.179	0.468	0.400
	p-value	< 0.001	-	0.023	< 0.001	< 0.001
SASP	Correlation coefficient	0.476	0.179	-	0.463	0.308
	p-value	< 0.001	0.023	-	< 0.001	< 0.001
SPA	Correlation coefficient	0.465	0.468	0.463	-	0.542
	p-value	< 0.001	< 0.001	< 0.001	-	< 0.001
SSP	Correlation coefficient	0.325	0.400	0.308	0.542	-
	p-value	< 0.001	< 0.001	< 0.001	< 0.001	-

**Table. 4:** Problem areas of Perception of Learning Environment among participants

Subscale	Domain Item	Mean (SD)
Perception of learning (SPL)	The teaching over emphasize factual learning	1.50 (0.75)
Perceptions of teaching (SPT)	The teachers ridicule the students	1.80 (0.92)
	The teachers are authoritarian	1.46 (0.85)
	The teachers provide constructive criticism here	1.68 (0.84)
	The teachers get angry in class	1.89 (1.07)
Academic self-perceptions (SASP)	I find the experience disappointing	1.70 (0.80)
Social self-perceptions (SSP)	I am too tired to enjoy this course	1.47 (1.00)
	I seldom feel lonely	1.76 (1.01)

Participating in extracurricular activities, travelling time to the faculty, and gender was observed as associated factors for the learning environment among BSc. Nursing undergraduates in state universities (Table 5).

**Table. 5:** Association of Perception of learning environment and its subscales with demographic characteristics

Variables	Categories	No. of	Sub domains (mean ± SD)				
		Participants	SPL	SPT	SASP	SPA	SSP
Age (Years)	22 - 24	61	30.7 (3.7)	25.9 (4.6)	22.8 (3.2)	29.6 (5.1)	16.6 (3.2)
	25 - 27	100	31.3 (4.1)	26.9 (4.2)	22.8 (3.3)	30.4 (4.9)	16.3 (3.0)
	p-value		NS	NS	NS	NS	NS
Gender	Male	50	30.2 (4.6)	25.8 (4.8)	23.2 (3.8)	30.6 (5.0)	16.7 (2.6)
	Female	111	31.7 (3.6)	26.9 (4.0)	22.6 (3.0)	29.9 (5.0)	16.3 (3.3)
	p-value		**0.045**	NS	NS	NS	NS
Living places	with parents	31	31.9 (3.0)	26.5 (3.6)	22.4 (3.6)	29.9 (3.4)	16.7 (3.1)
	rent/hostel	130	30.9 (4.1)	26.5 (4.4)	22.9 (3.2)	30.2 (5.3)	16.3 (3.1)
	p-value		NS	NS	NS	NS	NS
Transport	on foot	46	31.6 (3.3)	25.8 (4.6)	23.7 (3.1)	30.9 (5.7)	16.3 (3.0)
	by bus/vehicles	115	30.9 (4.2)	26.8 (4.1)	22.4 (3.3)	29.8 (4.7)	16.4 (3.1)
	p-value		NS	NS	**0.037**	NS	NS
Time to reach	< 30 min	120	30.8 (4.1)	26.5 (4.3)	22.7 (3.4)	29.8 (5.4)	16.3 (3.1)
to the University	≥ 30 min	41	31.9 (3.4)	26.5 (4.2)	23.1 (3.1)	31.2 (3.6)	16.9 (3.0)
	p-value		NS	NS	NS	NS	NS
part-time	Yes	13	30.1 (3.4)	26.0 (4.0)	22.8 (4.2)	32.2 (2.6)	16.3 (2.5)
employment	No	148	31.2 (4.0)	26.6 (4.3)	22.8 (3.2)	30.0 (5.1)	16.4 (3.1)
	p-value		NS	NS	NS	NS	NS
Extra professional	Yes	48	31.3 (4.0)	26.8 (3.9)	22.7 (3.5)	30.2 (5.4)	16.5 (3.5)
Course/ Diploma	No	113	31.0 (3.9)	26.4 (4.4)	22.8 (3.2)	30.2 (4.8)	16.4 (2.8)
	p-value		NS	NS	NS	NS	NS
Extra-curricular	Yes	75	31.8 (4.1)	26.6 (4.5)	23.4 (3.0)	30.6 (5.2)	16.8 (3.0)
activities	No	86	30.5 (3.8)	26.4 (4.0)	22.3 (3.5)	29.7 (4.8)	16.1 (3.1)
	p-value		**0.039**	NS	**0.034**	NS	NS

NS – Not significant

## Discussion

This study revealed that the overall mean score of student perception towards the learning environment was within the “more positive than negative” category in all the state universities. However, few problematic areas were observed in SPT, SASP, SSP, and SPL subdomains. Therefore, present findings revealed that the learning environment of Sri Lankan universities were one step behind the excellent category. It is suggested to address the problematic areas to reach the excellent category in the future. Furthermore, extracurricular activities, mode of transportation to faculty, and gender were observed as the associated factors for the learning environment. There were significantly higher mean scores of the learning environment in participating in extracurricular activities, by foot as mode transportation to the faculty and female gender, in comparison to those who did not participate in extracurricular activities, using other transportation mode and male gender.

Two Sri Lankan state universities; the University of Ruhuna and Eastern University have been conducted similar studies in the years 2012 and 2016 by using the DREEM questionnaire as a study tool to determine the learning environment. The result was revealed that the mean overall DREEM score were 109 and 111 in the University of Ruhuna and Eastern University respectively [13, 14]. However, the present study has also included both state universities which are named in arbitrary order A-F. The outcome of the study was, the mean overall DREEM scores were comparatively higher than the mean scores in previous studies in both universities. Therefore, considerable improvement has been taken placed in the learning environment during the last 5 years time.

Similar to present studies, recent studies conducted by few other countries revealed that the overall DREEM score was within the “more positive than negative” category [7, 17–20]. DREEM subdomain of SPL represented the “more positive perception” status in the present study and similar findings were observed in few other studies [7, 17, 18, 20]. However, Ahmed [19] revealed that SPL interpretation was “teaching is viewed negatively”. Furthermore, present results of SPT subdomain revealed that “moving to right direction” and a similar category was observed in few recent studies [7, 17–20]. The domain of SASP was the third domain and the findings revealed that it was within the “feeling more on the positive side” and Shrestha et al., [7], Gupta et al., [17], Barcelo et al., [18], and Ahmed et al., [19] and Ramsbotham et al., [20] also revealed the same finding among medical students. Subdomain of SPA represented within “more positive attitude” and that finding was similar to recent studies in India, Malaysia, Sudan Nepal, and Vietnam [7, 17–20]. SSP was the last subdomain of the DREEM, and present results were tally with other recent studies which was within the “not a bad” category [7, 17, 18, 20] but, Ahmed et al., [19] revealed that SSP was within the “not a nice place” category.

Furthermore, BSc. Nursing degree programs in Sri Lankan state universities are far better than in some Asian, African, and South American countries. The overall mean score of the DREEM questionnaire was 113, 112, and 106 among some medical faculties in Iran, Korea, and Kuwait in the years of 2019, 2015, and 2009 respectively [21–23]. Moreover, some African and South American countries such as Nigeria and Trinidad medicine programs were also observed lower mean scores when compared to the Sri Lankan nursing degree programs in state universities, however, these studies were carried out in the years 2001 and 2003. Therefore, the recent scores might be compatible with the Sri Lankan state universities [24, 25].

The nursing programs conducting in the South Asian regional countries such as Pakistan, Nepal, and Indonesia were identified that the all mean overall DREEM scores were just above 120 in the recent past and compatible with Sri Lankan status [25–29]. Furthermore, Australia also has shown compatible results in the field of dentistry when compared to the South Asian region BSc. Nursing degree programs [28]. Achieving a higher DREEM score may depend on more student-centered curricula, modified problem-based learning outcomes, and an effective combination of resources [29].

Furthermore, the few statements of SPL, SPT, SASP, and SSP subdomains have been identified as some problematic areas of perception of the learning environment in the current study. While the SPT subdomain was comparatively problematic in the current study, by 2012 the SASP subdomain was shown more problems among Sri Lankan nursing undergraduates [13, 14]. Similarly, Gupta et al., [17] revealed that the main problem area among Indian medical students in Government Medical College, Chandigarh was in SPT and SSP subdomains. Furthermore, Bakhshialiabad et al., [30] also stated that SPT and SSP subdomains were the main problem areas among medical sciences students in Iran. Therefore, it is essential to establish a stress-free learning environment with the aid of a supportive system to overcome these problematic areas [17, 18]. Furthermore, the process of academic activities needs to be concentrated on problem-solving approaches and improving the critical thinking abilities of students [17]. Additionally, addressing the accommodation and factual learning problems of students were also significant [30].

Gender, transportation mode, and participation in extra-curricular activities have been identified as associated factors of the learning environment in the present study. Furthermore, in the present study female undergraduates was observed a higher mean DREEM score in comparison to male undergraduates and that was similar to the DREEM score in Public School of Medicine, Brazil [9]. However, the studies conducted in India and Canada revealed that a lower DREEM score was observed among females when compared to male undergraduates [31, 32]. Additionally, factors such as age and monthly income were the other associated factors that were not significant in the present study [9]. Participating in extracurricular activities (sports, club activities) and living in a nearby university (mode of transportation) would be facilitated stress-free university life. Additionally, living nearby a university is also spared considerable time to engage with university academic activities. Therefore, consideration of these associated factors may help to overcome the problematic area of DREEM subdomains.

Nevertheless, there was some limitation in this study. The present results may affect the generalizability of the findings as the data collection has limited to final year students and comparatively small sample size and using a convenient sample. Further, using the interviewer administers tools for data collection can be enhanced the reliability of the results. Although limited demographic variables were assessed as the associated factors, there are many other predictors which are not included in this study such as cumulative academic performance of the students, number of failure subjects, number of hours engaging the academic activities in university (per week).

## Conclusions

All the BSc. Nursing degree programs in Sri Lankan state universities have shown that the overall learning environment was middle of the “more positive than the negative” category. Only SPL and SPT subdomains were identified as a considerable difference among state universities. However, nearly 15 years of nursing undergraduate history in Sri Lanka, none of the universities reaches the excellent category. Therefore, all the universities should consider their present status of the learning environment and need to address the problematic domains of the learning environment by considering the gaps in curricular and extracurricular activities in each university in future curricular revision.

## Data Availability

The datasets used and/or analyzed during the current study available from the corresponding author on reasonable request.

## Abbreviations

DREEM: The Dundee Ready Educational Environment Measure
SPL: Perception of learning
SPT: Perceptions of teaching
SASP: Academic self-perceptions
SPA: Perceptions of the atmosphere
SSP: Social self-perceptions
ANOVA: One-way Analysis of Variance
